# RLFDDA: a meta-path based graph representation learning model for drug–disease association prediction

**DOI:** 10.1186/s12859-022-05069-z

**Published:** 2022-12-01

**Authors:** Meng-Long Zhang, Bo-Wei Zhao, Xiao-Rui Su, Yi-Zhou He, Yue Yang, Lun Hu

**Affiliations:** 1 The Xinjiang Technical Institute of Physics and Chemistry, Chinese Academy of Sciences, Urumqi, China; 2 University of Chinese Academy of Sciences, Beijing, China; 3 Xinjiang Laboratory of Minority Speech and Language Information Processing, Urumqi, China; 4 School of Computer Science and Technology, Wuhan University of Technology, Wuhan, China

**Keywords:** Drug–disease association prediction, Meta-path based random walk, Graph representation learning, Heterogeneous network

## Abstract

**Background:**

Drug repositioning is a very important task that provides critical information for exploring the potential efficacy of drugs. Yet developing computational models that can effectively predict drug–disease associations (DDAs) is still a challenging task. Previous studies suggest that the accuracy of DDA prediction can be improved by integrating different types of biological features. But how to conduct an effective integration remains a challenging problem for accurately discovering new indications for approved drugs.

**Methods:**

In this paper, we propose a novel meta-path based graph representation learning model, namely RLFDDA, to predict potential DDAs on heterogeneous biological networks. RLFDDA first calculates drug–drug similarities and disease–disease similarities as the intrinsic biological features of drugs and diseases. A heterogeneous network is then constructed by integrating DDAs, disease–protein associations and drug–protein associations. With such a network, RLFDDA adopts a meta-path random walk model to learn the latent representations of drugs and diseases, which are concatenated to construct joint representations of drug–disease associations. As the last step, we employ the random forest classifier to predict potential DDAs with their joint representations.

**Results:**

To demonstrate the effectiveness of RLFDDA, we have conducted a series of experiments on two benchmark datasets by following a ten-fold cross-validation scheme. The results show that RLFDDA yields the best performance in terms of AUC and F1-score when compared with several state-of-the-art DDAs prediction models. We have also conducted a case study on two common diseases, i.e., paclitaxel and lung tumors, and found that 7 out of top-10 diseases and 8 out of top-10 drugs have already been validated for paclitaxel and lung tumors respectively with literature evidence. Hence, the promising performance of RLFDDA may provide a new perspective for novel DDAs discovery over heterogeneous networks.

## Background

Illness has always been a big problem plaguing people, and many people lose their lives due to diseases every day. In order to save more people’s lives, researchers have begun to study drugs for the treatment of various diseases [1–3]. Drugs can effectively relieve related symptoms caused by diseases, and ultimately achieve the goal of curing diseases. However, there will still be some sudden diseases, forcing human beings to continuously improve the efficiency of drug research and development. For example, the previous SARS virus and the current new coronavirus, both of which are sudden diseases, have strong transmission ability, and this requires researchers to develop corresponding drugs in a relatively short period of time [4, 5]. However, the development of a new drug takes a long period of time and consumes a lot of manpower and money [6]. Drug repositioning can effectively reduce the cost of drug research and development [7–9], and some existing computational models use the data related to drugs and diseases to predict unknown drug–disease associations (DDAs).

At present, there have been many studies that develop different prediction methods for drug repositioning. For methods based on machine learning, most of them take advantage of k-nearest neighbor (KNN), random forest (RF) and naive Bayes with features extracted from the biological information of drugs and diseases [10–16], and in this regard the task of drug repositioning can be considered as a binary classification problem. For example, PREDICT [10] integrates multiple drug–drug similarities and disease–disease similarities to construct drug and disease feature vectors, which are then taken as the input of a logistic regression classifier to predict unknown DDAs.

There are also deep learning-based methods [17–26], which use multilayer interconnected neuronal networks to transform the original features of drugs and diseases into high-level representations. However, they require a large amount of data for training, and also their performance needs to be fine-tuned accordingly for different training data. To obtain correlations between drugs and novel viruses, VDA-DLCMNMF [17] first uses the graph convolutional network to optimize the latent feature vectors of drugs and viruses, and then uses these feature vectors to calculate the correlation probabilities between drugs and viruses. Zhao et al. [18] use graph attention networks and graph embedding learning algorithms to learn local and global features of drug and disease nodes respectively, and achieve a high performance on benchmark datasets in terms of AUC.

Finally, network-based methods [27–39] are widely used for drug repositioning, as they are promising in learning feature representations of drugs and diseases from different networks for improved accuracy. Among them, heterogeneous networks are commonly adopted to represent the associations between different kinds of molecules. In particular, the nodes in a heterogeneous network can represent different types of molecules, and the edges can represent corresponding associations. For instance, deepDR [27] uses deep autoencoders to learn node representations from ten different heterogeneous networks, and uses collective variation autoencoders to predict potential DDAs. Although the above methods complete the task of DDA prediction well, they ignore the importance of other molecules that may contribute for better performance in discovering novel DDAs. Taking proteins as an example, disjointed drugs and diseases are possible to be connected through proteins, and their potential associations can thus be discovered. Meanwhile, most of network-based methods ignore the intrinsic structural characteristics of different molecules. Consequently, the latent knowledge in the network is difficult to be fully exploited for obtaining high-quality drug representation. There are similar studies that have considered the associations involving more than two biomolecule with molecular properties and network information. Yi et al. [40], integrate the associations between drug, protein, lncrna, miRNA, microbe, circRNA, mRNA and disease to form a molecular association network, and they use SDNE to learn the representations of nodes in the network. But they cannot be used well in heterogeneous complex relational networks that contain different kinds of nodes.

**Fig. 1:**
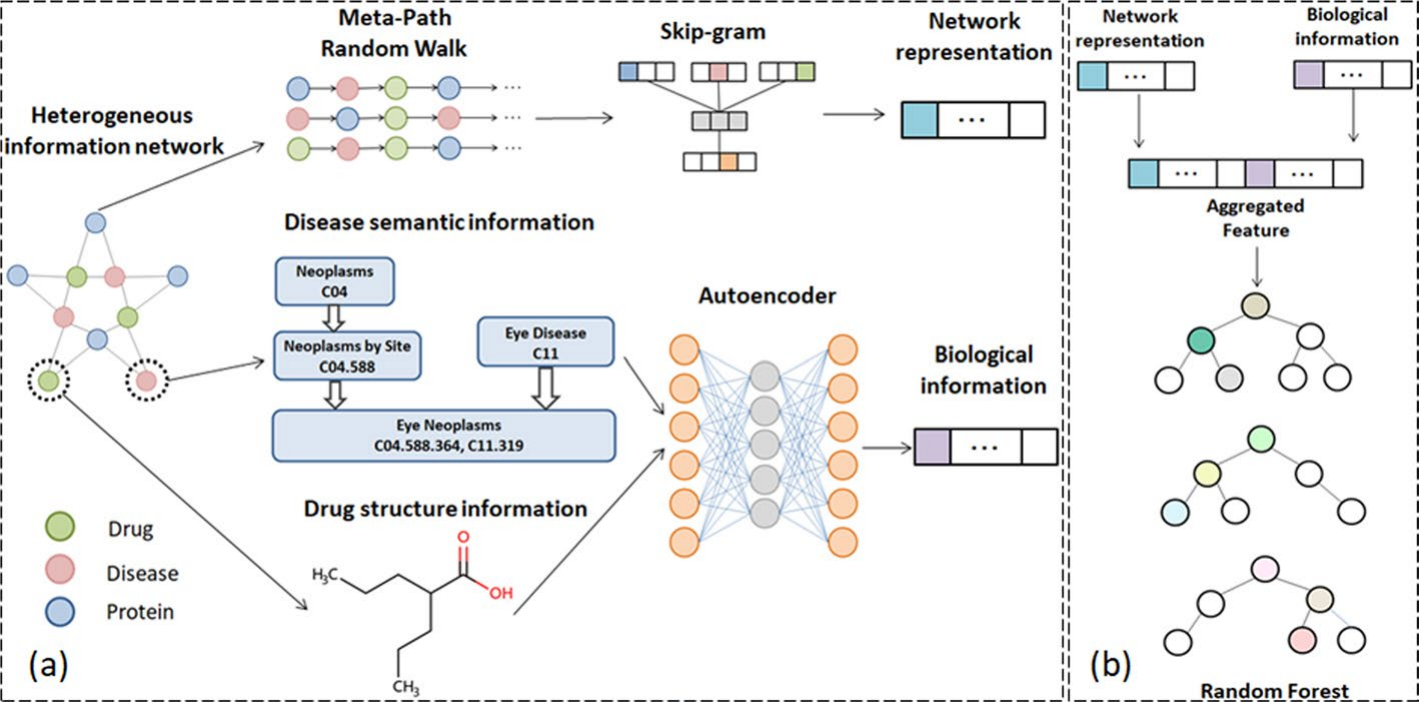
The overall workflow of RLFDDA, **a** the extraction of biological knowledge and network representations of drugs and diseases, **b** DDA prediction

To address the above challenges, in this paper, we propose a new model, namely RLFDDA, for DDA prediction by integrating the representations of different types of nodes in heterogeneous networks and the biological knowledge of the nodes themselves. To do so, RLFDDA first combines the drug–disease, disease–protein and drug–protein association networks into a heterogeneous information network. Then metapath2vec [41] is used to obtain the global representations of drugs and diseases. This is a method based on the meta-path random walking strategy. The method based on the meta-path random walking strategy can obtain a series of node sequences in a heterogeneous network by defining meta-paths, but it only takes into account the structural characteristics of nodes in the network, and does not consider additional information of nodes in the network. Therefore we additionally consider the biological information of drugs and diseases. In particular, the biological information of drugs is learned from their structures, and the biological information of diseases is obtained from their semantic knowledge graphs. Afterwards, the network representations of drugs and diseases, together with their own biological knowledge, are fused to construct their integrated feature representations, which are then used as the input to train a RF classifier. Last, potential DDAs can be predicted by the trained RF classifier. Experimental results show that our model achieves the best performance on two benchmark datasets under ten-fold cross-validation, as it outperforms several state-of-the-art prediction models in terms of independent evaluation metrics. The overall workflow of RLFDDA is shown in Fig. 1.

## Materials and methods

### Dataset

In this work, in order to better construct a heterogeneous information network, we use a benchmark dataset, i.e., B-dataset, containing three biological association networks, including drug–disease associations, disease–protein associations and drug–protein associations. Drug–disease associations in the B-dataset are obtained from the CTD database [42] by Zhang et al. [43], while drug–protein associations and disease–protein associations are collected from the DrugBank database [44] and DisGeNET database [45] respectively. Meanwhile, to evaluate the performance of the model, we use another benchmark dataset, i.e., C-dataset, which is collected by Luo et al. [46]. Some specific information about these two datasets are presented in Tables 1 and 2.

**Table 1:** Details of B-dataset

Heterogeneous network properties	Type	Number
Nodes	#Drug	269
	#Disease	598
	#Protein	1021
Associations	#Drug–disease	18416
	#Disease–protein	5898
	#Drug–protein	3110

**Table 2:** Details of C-dataset

Heterogeneous network properties	Type	Number
Nodes	#Drug	663
	#Disease	409
	#Protein	993
Associations	#Drug–disease	2532
	#Disease–protein	10734
	#Drug–protein	3773

### Biological knowledge extraction of drugs and diseases

When acquiring the biological knowledge of drugs, we find that SMILES (Simplified Molecular Input Line Entry System) [47] is used to represent the structure of drugs according to the Drugbank database. The standard SMILES is unique in that the names and structures of molecules are synonymous. Also SMILES can save storage space compared to two- and three-dimensional structures of molecules. Therefore, by analyzing the molecular structure of different drugs, we can discover potential relationships between drugs. Meanwhile, we use the RDKit [48] tool to obtain the specific chemical structures of drugs from SMILES. When the drug structure contains a specific component, the corresponding value is set to true, and false otherwise. Here we only consider a certain number of chemical structures, and after performing the above operation for each drug, we can obtain a matrix $R_{a}$ about the properties of drugs.

Regarding the similarity between diseases, we calculate its score by collecting medical subject descriptors of diseases from the MeSH (Medical Subject Headings) database [49], which provides a directed acyclic graph (DAG) with the descriptors to describe the diseases. Then we use the Jaccard formula to calculate the similarity between diseases. Taking the disease *r* as an example, we model its DAG as $DAG=(r,N_{r},E_{r})$, where $N_{r}$ represents the disease set associated with *r*, and $E_{r}$ represents the set of all links of *r*. Then the contribution of another disease *t* to *r* in *DAG* can be defined as:

1 $$\begin{aligned} \left\{ \begin{array}{ll} D_{r}(t)=1 &{}\quad if\; t=r \\ D_{r}(t)=\max \left\{ \gamma \cdot D_{r}(t^{'})|t^{'} \in children\ of\ t\right\} &{}\quad if \; t \not = r \end{array} \right. \end{aligned}$$

where $\gamma$ is the semantic contribution factor, and the semantic value can be obtained by summing the contribution values of all diseases in $N_{r}$. The formula is as follows [50]:

2 $$\begin{aligned} DV(r)=\sum _{t\in N_{r}}D_{r}(t) \end{aligned}$$

Combining Eqs. 1 and 2, the semantic similarity between diseases *t* and *r* can be calculated as:

3 $$\begin{aligned} sim(t,r)=\dfrac{\sum _{d\in N_{t} \cap N_{r}}(D_{t}(d)+D_{r}(d))}{DV(t)+DV(r)} \end{aligned}$$

where $D_{t} (d)$ and $D_{r} (d)$ represent the contributions of a disease *d* to *t* and *r* respectively. After calculating the similarity of all diseases, we can obtain the attribute feature matrix $D_{a}$ of diseases. $R_{a}$ and $D_{a}$ are then transformed into a more compact representation by using an autoencoder [51], which is a symmetric neural network model with input, hidden and output layers. The learning methods for both $R_{a}$ and $D_{a}$ are the same. Taking $R_{a}$ as an example, its learning function can be expressed as:

4 $$\begin{aligned} {(R_{a})^{'}}=\sigma (WR_{a}+b) \end{aligned}$$

where *b* is the bias, *W* is the weight matrix, and $\sigma (\cdot )$ is the activation function. After dimensionality reduction, we can get low-dimensional representations of drugs and diseases.

### Node representations of drugs and diseases

On the constructed heterogeneous information network, we can use the graph embedding method to learn the low-dimensional representations of nodes. We choose a method based on random walk. Metapath2vec is a meta-path-based random walk method proposed by Dong et al. [41], which can better capture the semantic and structural correlations between different nodes. To incorporate Metapath2vec, we first define a heterogeneous graph as $G=(V,E,T)$, where *V* represents the set of nodes, *E* represents the set of edges, and *T* represents the type of nodes or edges. In this study, the number of walks per node is 1000, the walk length is 100, the vector dimension is 64. Then according to the given meta-path *M*, the model performs random walk-based node sampling, where the transition probability at *i*-th step can be defined as:

5 $$\begin{aligned} P\left( {v^{i+1}|{v^{i}},M}\right) =\left\{ \begin{array}{ll} \dfrac{1}{{\left| N_{t+1}\left( v_{t}^{i}\right) \right| }}&{}\quad \left( v^{i+1},v_{t}^{i}\right) \in E,\; \emptyset (v^{i+1})=t+1 \\ 0 &{}\quad \left( v^{i+1},v_{t}^{i}\right) \in E,\; \emptyset (v^{i+1}) \not =t+1 \\ 0 &{}\quad \left( v^{i+1},v_{t}^{i}\right) \notin E \end{array} \right. \end{aligned}$$

Among them, $v_{t}^{i}\in V^{t}$ is the *i*-th node in the set of nodes with type *t*, $N_{t+1}(v_{t}^{i})$ indicates the number of nodes of type $t+1$ in the neighbors of $v_{t}^{i}$, and $\emptyset$ is a function that maps nodes to their types. After a meta-path-based random walk, we can get a path containing drug and disease nodes, and use this path as the input to the skip-gram model [52] to generate the low-dimensional representations of nodes. The skip-gram model is usually used to predict the word of the context according to the current word. It usually contains three layers or more, and is divided into two parts. The first part is the establishment of the model and the second part is the acquisition of the word embedding vector. Its input is usually in the form of one-hot, and the parameters in the network are learned through training. The objective function for a skip-gram model is:

6 $$\begin{aligned} L=\dfrac{1}{T}\sum _{t=1}^{T}\sum _{-c\le j \le c,j\not =0} \log {p(w_{t+j}|w_{t})} \end{aligned}$$

where *c* is the sliding window size, *T* is the total number of nodes, $w_{(t+j)}$ and $w_{t}$ represent the $(t+j)$-th node and the *t*-th node. Regarding *c*, if its value becomes larger, the accuracy is improved with more samples trained. The skip-gram model uses the softmax function to define $p(w_{j} |w_{i})$ as:

7 $$\begin{aligned} p(w_{j} | w_{i})=\dfrac{\exp \left( v_{w_{j}}^{' T} v_{w_{i}}\right) }{\sum _{w=1}^{W} v_{w_{j}}^{' T} v_{w_{i}}} \end{aligned}$$

where $v_{w}^{'}$ and $v_{w}$ denote the output and input vectors respectively. By using the above two methods, we can obtain the representations of drug and disease nodes in the network.

### DDA prediction

After obtaining the representations of drugs and diseases, the aggregation function LMF [53] is used to fuse these two representations in order to obtain their final representations. Since the feature fusion methods of drugs and diseases are the same, the feature fusion of drugs is taken as an example. Specifically, assuming that the attributes of drug node *i* and the network nodes are $R_{a}^{i}$ and $R_{e}^{i}$, we first convert them to two tensors $z_{a}$ and $z_{e}$ respectively. The final representation of *i* is defined as:

8 $$\begin{aligned} R^{i}=\sigma \left( \sum _{i=1}^{r}W_{a}^{i}\cdot z_{a} + \sum _{i=1}^{r}W_{e}^{i}\cdot z_{e}\right) +b \end{aligned}$$

where $R^{i}$ represents the final representation of *i*, $\sum _{i=1}^{r}W_{a}^{i}$ and $\sum _{i=1}^{r}W_{e}^{i}$ are the rank *r* factorization of $z_{a}$ and $z_{e}$ respectively, *r* is a constant value, and *b* is the bias. In this study, predicting potential DDAs is a binary task. Hence, for a given drug–disease pair, we input their final representations to a RF classifier to predict the existence of an association between them.

## Results and discussion

### Performance evaluation metrics

For the two benchmark datasets B-dataset and C-dataset that we use to construct heterogeneous information networks, the specific information contained in them can be found in Tables 1 and 2. All known DDAs in these two benchmark datasets are considered as positive samples, and we then generate the set of negative samples with an equal size. When generating negative samples, we randomly pair up drugs and diseases whose associations are not found in the positive samples.

To validate the performance of RLFDDA on two benchmark datasets, we use ten-fold cross-validation, which randomly divides the dataset into ten folds. Each fold is alternatively selected as the test set, and the rest are used as the training set. We then repeat the validation process for ten times and take the average score of each metric as the final performance of the model. To quantify the results of ten-fold cross-validation, we use several evaluation criteria, including accuracy (Acc.), precision (Prec.), recall (Recall), the area under ROC curve (AUC) and F1-score, such that the quality, robustness and predictability of the model can be measured from different perspectives. The relevant definitions are as below.

9 $$\begin{aligned} Acc.= & {} \dfrac{TN+TP}{FP+TP+FN+TN} \end{aligned}$$

10 $$\begin{aligned} Prec.= & {} \dfrac{TP}{FP+TP} \end{aligned}$$

11 $$\begin{aligned} Recall= & {} \dfrac{TP}{FN+TP} \end{aligned}$$

12 $$\begin{aligned} F1-score= & {} \dfrac{2*Prec*Recall}{Prec+Recall} \end{aligned}$$

In the above definitions, FP, TP, FN and TN respectively represent the numbers of false positive, true positive, false negative and true negative samples respectively.

### Evaluate prediction performance

In this section, we conduct ten-fold cross-validation on two benchmark datasets, B-dataset and C-dataset, to evaluate the performance of our model. The performance on each fold and the overall performance of RLFDDA are presented in Tables 3 and 4. From this we can see that the values of Acc., AUC, Prec., recall and F1-score on the B-dataset are 0.7907, 0.8728, 0.7821, 0.8060 and 0.7938 respectively. Regarding the standard deviation value of each metric, it is 0.0061 on Acc., 0.0063 on AUC, 0.0084 on Prec., 0.0078 on recall, and 0.0057 on F1-score. For C-dataset, the values of Acc., AUC, Prec., recall and F1-score are 0.9006, 0.9636, 0.9035, 0.8972 and 0.9002 respectively. At the same time, the standard deviation values of each metric are 0.0121, 0.0047, 0.0136, 0.0222 and 0.0129 respectively. These results demonstrate the good performance of the model.

**Table 3:** The performance of RLFDDA on each fold in cross-validation over B-dataset

Fold	Acc.	AUC.	Prec.	Recall	F1-score
0	0.7858	0.8673	0.7707	0.8138	0.7917
1	0.7964	0.8727	0.7939	0.8008	0.7973
2	0.7972	0.8785	0.7889	0.8116	0.8001
3	0.7932	0.8760	0.7807	0.8154	0.7977
4	0.7796	0.8639	0.7691	0.7991	0.7838
5	0.7904	0.8753	0.7843	0.8013	0.7927
6	0.7959	0.8813	0.7927	0.8013	0.7970
7	0.7872	0.8702	0.7747	0.8100	0.7919
8	0.7831	0.8628	0.7784	0.7915	0.7849
9	0.7978	0.8802	0.7876	0.8154	0.8013
Overall	0.7907 ± 0.0061	0.8728 ± 0.0063	0.7821 ± 0.0084	0.8060 ± 0.0078	0.7938 ± 0.0057

**Table 4:** The performance of RLFDDA on each fold in cross-validation over C-dataset

Fold	Acc.	AUC.	Prec.	Recall	F1-score
0	0.8996	0.9610	0.8980	0.9016	0.8998
1	0.8917	0.9643	0.9129	0.8661	0.8889
2	0.9114	0.9721	0.9300	0.8898	0.9095
3	0.8701	0.9544	0.8821	0.8543	0.8680
4	0.9075	0.9613	0.8966	0.9213	0.9087
5	0.9055	0.9610	0.9187	0.8898	0.9040
6	0.9055	0.9674	0.8992	0.9134	0.9063
7	0.8957	0.9663	0.8972	0.8937	0.8955
8	0.9035	0.9678	0.8897	0.9213	0.9052
9	0.9154	0.9622	0.9105	0.9213	0.9159
Overall	0.9006 ± 0.0121	0.9636 ± 0.0047	0.9035 ± 0.0136	0.8972 ± 0.0222	0.9002 ± 0.0129

### Impact of various feature type

**Fig. 2:**
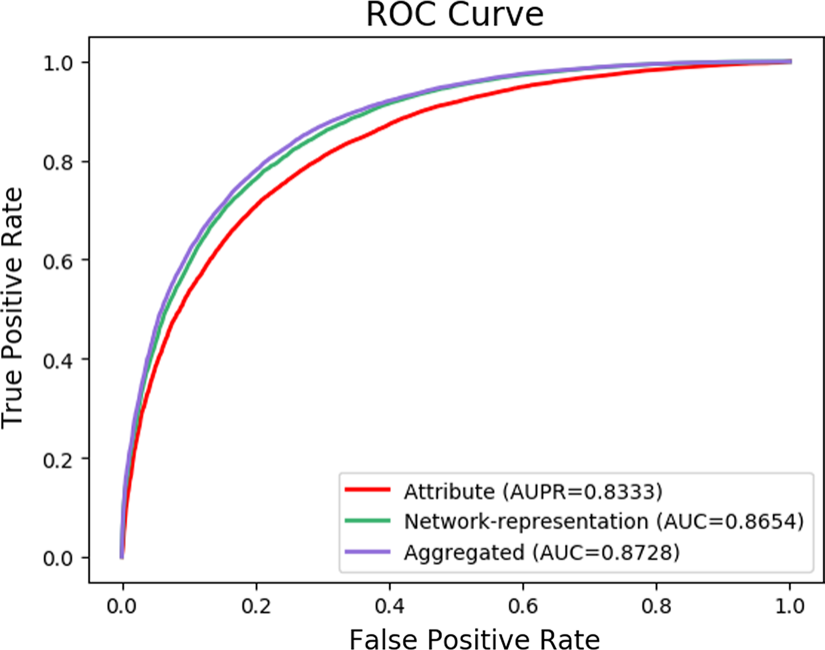
The ROC curve of attribute, network and aggregated features on B-dataset

**Fig. 3:**
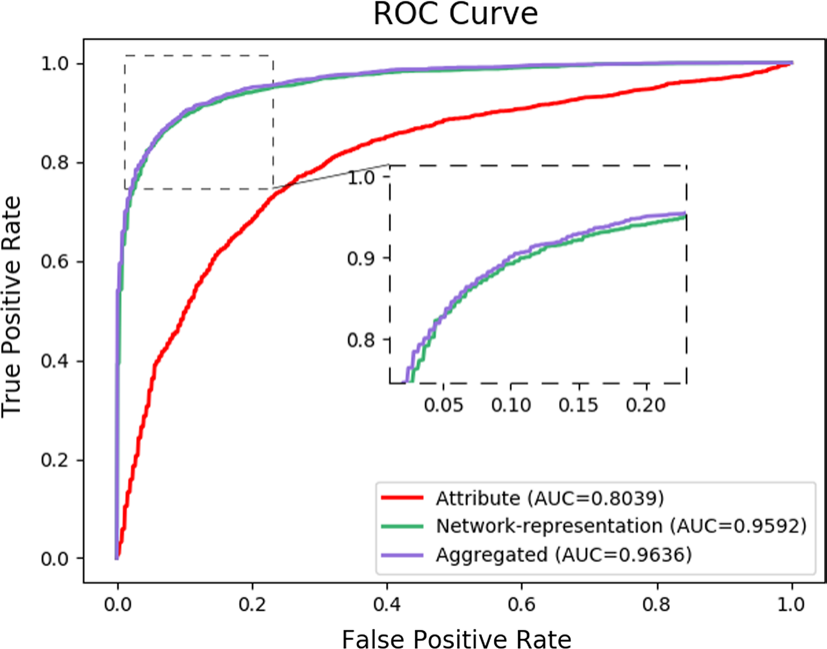
The ROC curve of attribute, network and aggregated features on C-dataset

As mentioned above, RLFDDA takes into account not only the biological knowledge of drugs and diseases, but also their network representations when making a prediction. In this section, we design two variants to perform separate analyses on these two characteristics. More specifically, the first variant only considers the biological knowledge of drugs and diseases, while the second only considers their network representation to predict DDAs. From Table 5, Figs. 2 and 3, we can observe that the best results can be obtained by aggregating these two kinds of features. When only considering the biological knowledge of drugs and diseases, RLFDDA obtains the worst performance on all evaluation metrics, indicating that only considering the biological knowledge is not able to predict the potential DDAs accurately. Compared with the variant only considering the biological information of drugs and diseases, the network representations of drugs and diseases obtained from the heterogeneous network improve the performance in all indicators, indicating that heterogeneous network information can help us predict potential DDAs.

**Table 5:** Experimental results of two variants of RLFDDA

Dataset	type	Acc.	AUC.	Prec.	Recall	F1-score
B-dataset	Attribute	0.7555 ± 0.0060	0.8333 ± 0.0063	0.7495 ± 0.0078	0.7676 ± 0.0079	0.7584 ± 0.0056
	Network	0.7823 ± 0.0055	0.8654 ± 0.0044	0.7756 ± 0.0071	0.7945 ± 0.0082	0.7849 ± 0.0054
	Aggregated	0.7907 ± 0.0061	0.8728 ± 0.0063	0.7821 ± 0.0084	0.8060 ± 0.0078	0.7938 ± 0.0057
C-dataset	Attribute	0.7482 ± 0.0131	0.8039 ± 0.0154	0.7521 ± 0.0166	0.7413 ± 0.0226	0.7464 ± 0.0138
	Network	0.8961 ± 0.0103	0.9592 ± 0.0094	0.9023 ± 0.0113	0.8886 ± 0.0218	0.8952 ± 0.0112
	Aggregated	0.9006 ± 0.0121	0.9636 ± 0.0047	0.9035 ± 0.0136	0.8972 ± 0.0222	0.9002 ± 0.0129

### Performance comparison

**Fig. 4:**
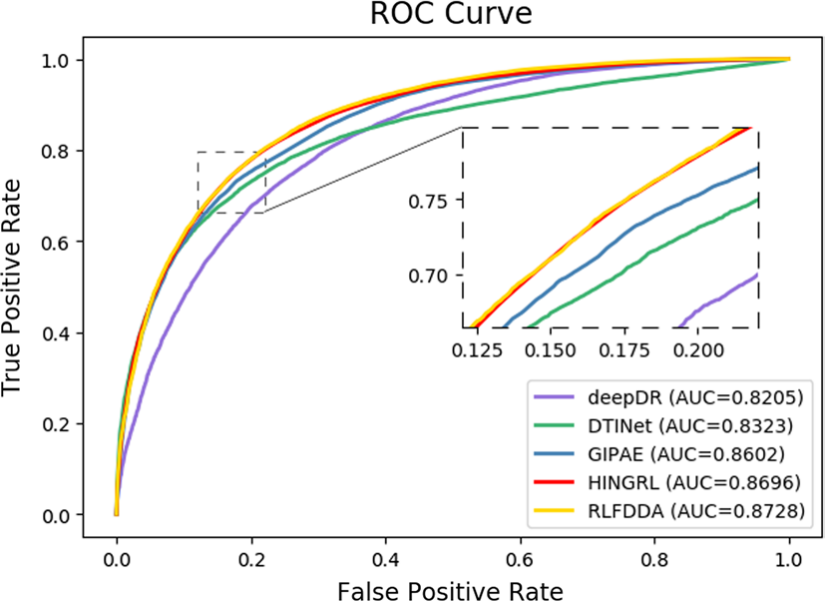
The ROC curves of all algorithms on B-dataset

**Fig. 5:**
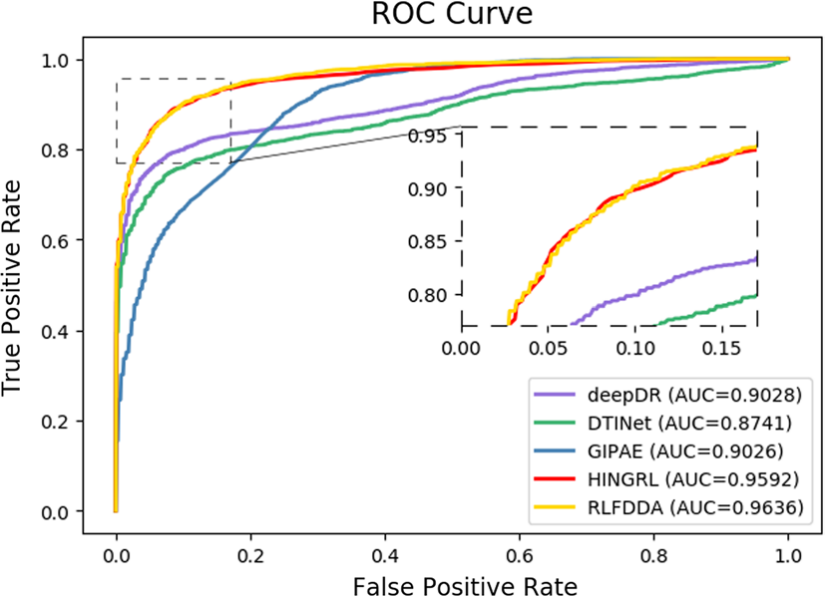
The ROC curves of all algorithms on C-dataset

To better evaluate the performance of RLFDDA, we compare RLFDDA with several state-of-the-art prediction models, including deepDR [27], DTINet [54], GIPAE [55] and HINGRL [28] on two benchmark datasets. In particular, DTINet uses a computational pipelines to learn the low-dimensional feature vector representations from multiple drug–related heterogeneous information networks and predict potential drug–target associations. GIPAE uses autoencoder and Gaussian interaction contour kernel to obtain the feature descriptors of drugs and diseases for predicting DDAs. HINGRL predicts DDAs by using deepwalk to obtain network representations of drugs and diseases, which are then fused with their own attributes. The results are shown in Figs. 4 and 5 and Table 6. RLFDDA achieves higher AUC values on the two datasets, which are 0.32%, 1.26%, 4.05% and 5.23% better than HINGRL, GIPAE, DTINet and deepDR on the B-dataset, respectively, 0.44%, 6.1%, 8.95%, and 6.08% better on the C-dataset, respectively. Another point worth point is the performances of deepDR and DTINet on the B-dataset, as they obtain larger Prec. values, but their Recall values are lower. This indicates that although these methods can accurately detect positive samples, they have serious missed detections. In the C-dataset, GIPAE has a higher Recall value and a lower Prec. value, indicating that it has fewer missed detections but with a higher false detection rate. There is a conflict between precision and recall. Therefore, in order to comprehensively consider the prediction performance, we use the metric of F1-score. It is the summed average of precision and recall, and the F1-score considers recall and precision to be equally important for binary classification problems. We can see that RLFDDA achieves the largest F-score values on both the B-dataset and C-dataset benchmark datasets. This could also a strong indicator for the good performance of RLFDDA.

**Table 6:** Experimental results of performance comparison on two benchmark datasets

Dataset	Methods	AUC.	Prec.	Recall	F1-score
B-dataset	deepDR	0.8205	0.8813	0.2345	0.3704
	DTINet	0.8323	**0.9712**	0.1781	0.3009
	GIPAE	0.8602	0.7869	0.7768	0.7788
	HINGRL	0.8696	0.7875	0.7953	0.7913
	RLFDDA	**0.8728**	0.7821	**0.8060**	**0.7938**
C-dataset	deepDR	0.9028	0.9894	0.5450	0.7021
	DTINet	0.8741	**0.9974**	0.1376	0.2409
	GIPAE	0.9026	0.7619	**0.9202**	0.8336
	HINGRL	0.9592	0.9058	0.8878	0.8966
	RLFDDA	**0.9636**	0.9035	0.8972	**0.9002**

Best results are bolded

### Impact of different classifiers

**Fig. 6:**
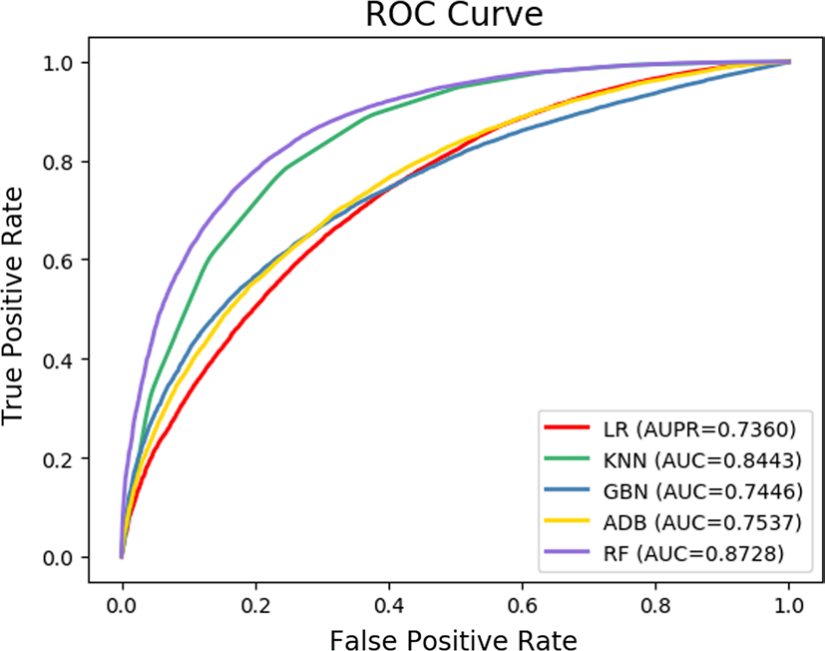
The ROC curves of RLFDDA by using different classifiers on B-dataset

**Fig. 7:**
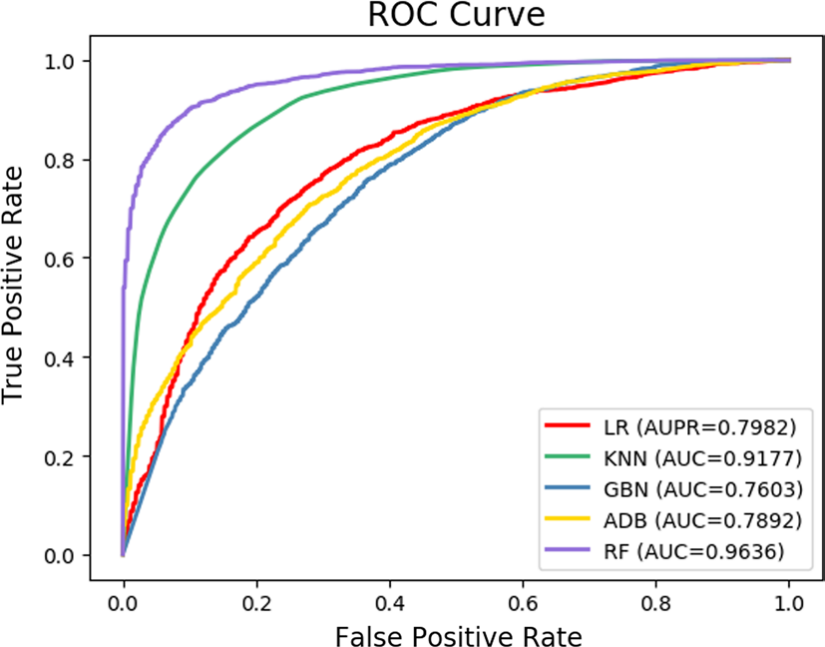
The ROC curves of RLFDDA by using different classifiers on C-dataset

In this section, we evaluate the effectiveness of classifiers adopted by the model. The RF classifier is applied to complete the prediction task by RLFDDA, and achieves good results. In order to better show the effectiveness of RF, we use some other classifiers to replace it for making a fair comparison. The classifiers used include AdaBoost (ADB), Gaussian Naive Bayes (GNB), K-Nearest Neighbor (KNN) and Logistic Regression (LR). We keep other parameters in the model unchanged, and use the same dataset for training and testing. The parameters used by the above four classifiers are all the default parameters, and the specific experimental results are presented in Tables 7 and 8 and Figs. 6 and 7. From these tables and figures, we can see that RF achieves good results in terms of Acc. and AUC. Moreover, we note that KNN obtains better results on Recall but its Prec. values are low, indicating that KNN has a low probability of missed detection, but a high probability of false detection. Regarding F1-score, we can see that RF has higher F1-scores on B-dataset and C-dataset, indicating the rationality behind the use of RF.

**Table 7:** Experimental results of different classifiers on B-dataset

Classifier	Acc.	AUC.	Prec.	Recall	s F1-score
AdaBoost	0.6871 ± 0.0073	0.7537 ± 0.0081	0.6825 ± 0.0070	0.6995 ± 0.0121	0.6909 ± 0.0081
GNB	0.6814 ± 0.0067	0.7446 ± 0.0059	0.6774 ± 0.0062	0.6927 ± 0.0111	0.6849 ± 0.0077
KNN	0.7230 ± 0.0036	0.8443 ± 0.0064	0.6538 ± 0.0028	**0.9480** ± **0.0052**	0.7739 ± 0.0029
LR	0.6705 ± 0.0070	0.7360 ± 0.0082	0.6689 ± 0.0072	0.6752 ± 0.0095	0.6721 ± 0.0074
RF	**0.7907** ± **0.0061**	**0.8728** ± **0.0063**	**0.7821** ± **0.0084**	0.8060 ± 0.0078	**0.7938** ± **0.0057**

Best results are bolded

**Table 8:** Experimental results of different classifiers on C-dataset

Classifier	Acc.	AUC.	Prec.	Recall	F1-score
AdaBoost	0.7063 ± 0.0169	0.7892 ± 0.0147	0.7249 ± 0.0196	0.6657 ± 0.0258	0.6938 ± 0.0185
GNB	0.6787 ± 0.0202	0.7603 ± 0.0177	0.6942 ± 0.0187	0.6382 ± 0.0301	0.6649 ± 0.0243
KNN	0.8293 ± 0.0150	0.9177 ± 0.0196	0.7759 ± 0.0136	**0.9264** ± **0.0173**	0.8444 ± 0.0136
LR	0.7348 ± 0.0261	0.7982 ± 0.0221	0.7316 ± 0.0260	0.7421 ± 0.0338	0.7366 ± 0.0270
RF	**0.9006** ± **0.0121**	**0.9636** ± **0.0047**	**0.9035** ± **0.0136**	0.8972 ± 0.0222	**0.9002** ± **0.0129**

Best results are bolded

### Case study

In order to better demonstrate the model’s ability to predict the potential DDAs, we use B-dataset as the training data of our model. With the trained model, we predict the top-10 diseases related to paclitaxel and top-10 drugs related to lung neoplasms. Any existing associations between drugs and diseases in the data set are not used when we make a prediction with RLFDDA. After that, we investigate the prediction results, and select some relevant cases for analysis. As can be seen from Table 9, among top-10 predicted diseases related to paclitaxel, seven of them have been verified by relevant literature. Taking acute kidney injury as an example. Xu et al. [56] point out that paclitaxel can reduce acute kidney injury induced by lipopolysaccharide that regulates lnc-MALAT 1/mi-R 370-3 p/HMGB 1 axis and the expression of TNF-$\alpha$, IL-6 and IL-1 $\beta$. There are many reasons for kidney damage, such as heart disease and vascular inflammation. These two diseases are also predicted in the prediction of paclitaxel-related diseases, so we have reason to believe that this model can predict potential DDAs.

**Table 9:** Top-10 diseases predicted to be associated with paclitaxel

Drug	Disease	MESH ID	Score	Evidence (PMID)
Paclitaxel	Cardiovascular diseases	D002318	0.9449	PMID: 16239414
	Colonic neoplasms	D003110	0.9339	PMID: 31701488
	Acute kidney injury	D058186	0.9289	PMID: 32998017
	Inflammation	D007249	0.9259	PMID: 23318472
	Fibrosis	D005355	0.9259	PMID: 33749060
	Arrhythmias, cardiac	D001145	0.9219	PMID: 33624748
	Proteinuria	D011507	0.9129	N/A
	Liver diseases	D008107	0.8999	PMID: 27043783
	Anxiety disorders	D001008	0.8908	N/A
	Weight loss	D015431	0.8798	N/A

Lung neoplasms are usually associated with viral infections, mycotoxins, smoking, etc. From Table 10, we note that top-10 drugs predicted by the model have therapeutic effects on lung neoplasms. It can be seen that among top-10 drugs, eight of them have positive therapeutic effects on lung neoplasms, and they have been verified by relevant literature.

**Table 10:** Top-10 drugs predicted to be associated with Lung neoplasms

Disease	Drug	DrugBank ID	Score	Evidence(PMID)
Lung neoplasms	Ibuprofen	DB01050	0.8508	PMID: 15756426
	Prednisone	DB00635	0.8438	PMID: 34853306
	Acetaminophen	DB00316	0.8238	PMID: 31541463
	Tretinoin	DB00755	0.7797	PMID: 31456481
	Valproic acid	DB00313	0.7778	PMID: 32290325
	Daunorubicin	DB00694	0.7727	PMID: 31239668
	Carbamazepine	DB00564	0.7538	N/A
	Sulfasalazine	DB00795	0.7537	PMID: 19104813
	Diethylstilbestrol	DB00255	0.7257	PMID: 34281152
	Hydrocortisone	DB00741	0.7247	N/A

### Independent dataset validation experiment

In order to further verify the ability of RLFDDA, we use an additional dataset, F-dataset, for independent validation. This dataset is collected by Gottlieb et al. [10], and it contains 592 drugs, 313 diseases and 1933 DDAs. In particular, we take the drug–disease associations contained in the C-dataset as positive samples, and generate negative samples with an equal size. Then we combine all positive and negative samples to compose the training set. DDAs in the F-dataset are regarded as positive samples in the test set, and we also generate negative samples with an equal size in the test set. It is worth noting that C-dataset and F-dataset should share certain drugs and diseases. In the experimental results, the Acc., F1-score and AUC scores obtained by RLFDDA are 0.9240, 0.9276 and 0.9912 respectively. In addition, we also use the F-dataset as the training set and the C-dataset as the test set. The Acc., F1-score and AUC scores obtained by RLFDDA are 0.7350, 0.7709 and 0.9054 respectively. The experimental results show that RLFDDA still performs well in independent validation, thus having strong generalization ability.

## Conclusion

In this work, we propose a new model, namely RLFDDA, for predicting potential DDAs. By integrating the associations between drugs, diseases and proteins, we construct a heterogeneous network and use meta-path-based graph representation learning to capture the features of drugs and diseases. The acquired features are then fused with their own biological knowledge to obtain the final representations of drugs and diseases. RLFDDA finally uses a RF classifier to predict potential DDAs. Our experimental results show that RLFDDA achieves good results on all benchmark datasets and outperforms several state-of-the-art methods. Two case studies of paclitaxel and lung neoplasms show that RLFDDA has excellent performance in predicting potential DDAs. There are some limitations about the performance of RLFDDA. First, the sample size of the selected dataset is limited and only proteins are considered as intermediate molecules. RLFDDA also requires to manually design meta-paths, and this fact is difficult to achieve optimal performance in practice. In future work, we intend to integrate more kinds of biomolecules into the network and evaluate the importance of these molecules in the task of DDA prediction. We are also interested in using RLFDDA for other related applications, such as protein–protein interactions [57] and associations between circRNA and diseases [58].

## Data Availability

The datasets used and/or analysed during the current study available from the corresponding author on reasonable request.
